# Retraction statement: The pattern of neurological manifestations of tuberculosis among adult Sudanese patients attending multi‐neurological centers and hospitals in Sudan: A hospital‐based cross‐sectional study

**DOI:** 10.1002/hsr2.1529

**Published:** 2023-08-24

## Abstract

Etedal Ahmed A. Ibrahim, Nosiba Ibrahim Mohammed Ahmed Hamza Mohammed, Khabab Abbasher Hussien Mohamed Ahmed, Gaffar Alemam A. Manhal, Mohammed Mahmmoud Fadelallah Eljack, Muhammad Junaid Tahir, HSR21068, https://doi.org/10.1002/hsr2.1068.

The above article, published online on {First published: 19 January 2023} in Wiley Online Library {https://onlinelibrary.wiley.com/doi/full/10.1002/hsr2.1068} has been retracted by agreement between the journal's Editor‐in‐Chief, Charles Young and John Wiley & Sons Ltd. The retraction has been agreed given the journal has received evidence confirming that the peer review process of this paper was manipulated. As a result, the conclusions reported in the article are not considered reliable.

Etedal Ahmed A. Ibrahim, Nosiba Ibrahim Mohammed Ahmed Hamza Mohammed, Khabab Abbasher Hussien Mohamed Ahmed, Gaffar Alemam A. Manhal, Mohammed Mahmmoud Fadelallah Eljack, Muhammad Junaid Tahir, HSR21068, https://doi.org/10.1002/hsr2.1068.

The above article, published online on {First published: 19 January 2023} in Wiley Online Library {https://onlinelibrary.wiley.com/doi/full/10.1002/hsr2.1068} has been retracted by agreement between the journal's Editor‐in‐Chief, Charles Young and John Wiley & Sons Ltd. The retraction has been agreed given the journal has received evidence confirming that the peer review process of this paper was manipulated. As a result, the conclusions reported in the article are not considered reliable.

